# Enhanced Sensitivity of Anti-Symmetrically Structured Surface Plasmon Resonance Sensors with Zinc Oxide Intermediate Layers

**DOI:** 10.3390/s140100170

**Published:** 2013-12-20

**Authors:** Yi-Chen Tu, Teng-Yi Huang, Nan-Fu Chiu

**Affiliations:** Institute of Electro-Optical Science and Technology, National Taiwan Normal University, No. 88, Sec. 4, Ting-Chou Road, Taipei 11677, Taiwan; E-Mails: loveregg1203@yahoo.com.tw (Y.-C.T.); tyhuang1120@gmail.com (T.-Y.H.)

**Keywords:** intermediary layer, surface plasmon resonance (SPR), zinc oxide (ZnO), full width at half maximum (FWHM)

## Abstract

We report a novel design wherein high-refractive-index zinc oxide (ZnO) intermediary layers are used in anti-symmetrically structured surface plasmon resonance (SPR) devices to enhance signal quality and improve the full width at half maximum (FWHM) of the SPR reflectivity curve. The surface plasmon (SP) modes of the ZnO intermediary layer were excited by irradiating both sides of the Au film, thus inducing a high electric field at the Au/ZnO interface. We demonstrated that an improvement in the ZnO (002) crystal orientation led to a decrease in the FWHM of the SPR reflectivity curves. We optimized the design of ZnO thin films using different parameters and performed analytical comparisons of the ZnO with conventional chromium (Cr) and indium tin oxide (ITO) intermediary layers. The present study is based on application of the Fresnel equation, which provides an explanation and verification for the observed narrow SPR reflectivity curve and optical transmittance spectra exhibited by (ZnO/Au), (Cr/Au), and (ITO/Au) devices. On exposure to ethanol, the anti-symmetrically structured showed a huge electric field at the Au/ZnO interface and a 2-fold decrease in the FWHM value and a 1.3-fold larger shift in angle interrogation and a 4.5-fold high-sensitivity shift in intensity interrogation. The anti-symmetrically structured of ZnO intermediate layers exhibited a wider linearity range and much higher sensitivity. It also exhibited a good linear relationship between the incident angle and ethanol concentration in the tested range. Thus, we demonstrated a novel and simple method for fabricating high-sensitivity, high-resolution SPR biosensors that provide high accuracy and precision over relevant ranges of analyte measurement.

## 1. Introduction

The surface plasmon resonance (SPR) behavior of free electrons or plasma at the interface of a metal-dielectric material has been widely studied [1–4]. The techniques of attenuated total reflection (ATR) prism coupler-based SPR between Kretschmann and Otto configurations have been used extensively to study the plasmonic material properties for specific frequencies and measurement applications, including refractive index (*n*), extinction coefficient (*k*), thickness (*d*), and roughness (*r*) [5,6]. Conventionally, SPR biosensors are used in biochemistry and biology to detect molecular concentration, thickness, and specific chemistry analytes [7,8]. In biochemistry, analyte concentration is determined from the SPR angle shift by a biosensor operating in the angular interrogation mode. The shift or difference between the initial and final values of the SPR angles provides a quantitative measurement of the analyte concentration. A prism-based SPR sensor is used in the conventional ATR method; these conventional SPR sensors generally consist of gold (Au) deposited on either a chromium (Cr) or titanium (Ti) adhesion layers (2–5 nm). For light with a wavelength of 632 or 658 nm, the Cr/Au and Ti/Au films exhibit low-sensitivity with large full width at half maximum (FWHM) values of approximately 3° [9–11]. However, these conventional SPR sensors (Cr/Au) can cause problems in the adhesion layer, such as metal interdiffusion, low optical transmission, large FWHM, and a reduction in biosensing sensitivity [12,13]. In addition, several different SPR device configurations have been shown to exhibit improved plasmon emission efficiency, such as devices showing active plasmon-coupled emission [14], prism-based couplers with periodic metallic nanostructures [15], and multilayer devices [16]. Recently, high-refractive-index germanium (Ge) semiconductor films [17], indium-tin-oxide (ITO) transparent conducting films [18] and titanium nitride (TiN_x_) adhesion layers [19] have been reported to show improved SPR performance characteristics.

In this study, we have developed a method based on the plasmonic structures that can help to increase the detection sensitivity, resolution, response time, accuracy and improve the performance of SPR biosensors. As a semiconductor material, ZnO thin films exhibit excellent optical and electrical properties, including a high refractive index and high transparency [20,21]. The anti-symmetrically structured should be extended concerning the possible application of the studies also for the different kind photo induced and nonlinear optical effects. In this case besides the plasmons additional role on ZnO/Au structures begin to play phonons interacting with the nano-trapping levels [22]. Many studies have explored the fabrication of ZnO nanostructures using Au nanoparticles [23–26], because ZnO thin films enhance the optical properties of SPR devices. The framework of plasmonic studies have demonstrated the ability of the asymmetric structures to provide qualitative or quantitative information, but the evaluation of their sensitivity as compared to conventional SPR methods has not been broadly investigated.

In our previous study, we demonstrated the detection of carbohydrate antigen (CA) 15-3, a tumor marker for breast cancer, using a Au/ZnO SPR device that offers highly sensitive detection of biomarkers [27]. In the present study, we fabricated an intermediary ZnO layer for the theoretical analysis of anti-symmetrically structured SPR devices. It is shown that an improvement in the ZnO (002) crystal orientation led to a decrease in the FWHM of the SPR reflectivity curves. As a proof of the concept, we show the possibility of anti-symmetric structure characterization of some semiconductor-based films using the newly introduced ZnO-based technology. Furthermore, we determine the optimal thickness of the ZnO and Au thin-film layers in the anti-symmetric structures to improve the SPR efficiency, induce a high electric field and obtain a narrow SPR reflectivity curve.

## 2. Materials and Methods

### 2.1. Model of the Anti-Symmetrically Structured SPR Biosensors

**Figure 1.**
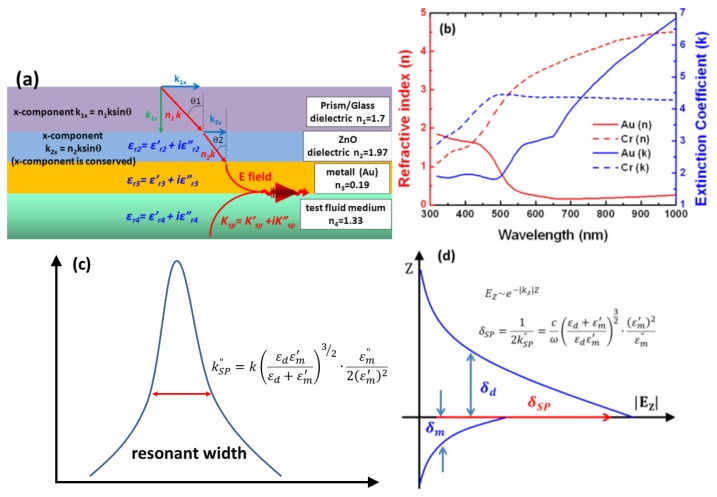
(**a**) Anti-symmetric structure of a four-layered SPR system consisting of an SF10 prism substrate, high-refractive-index ZnO intermediary layer, gold film, and test fluid medium. *K_sp1_* and *K_sp2_* denote the wave propagation number along the *x* axis for the evanescent wave and the SPR wave, respectively. The incident angle at which the TM wave of the evanescent field wave-vector matches the surface plasmon wave-vector is called the resonance angle, *θ*_SPR_. (**b**) UV-vis-NIR spectra to determine the effective refractive index (**n** = *n* + *ik*), the real part (*n*) of the refractive index, and the imaginary part of the extinction coefficient (*k*) of dielectric function of Au with Cr. The FWHM of the SPRs is dependent on the real and imaginary parts of the complex dielectric constant of gold. A reduction in the FWHM of the SPR implies an increase in the propagation length of the SPPs. (**c**) is SPR resonance width and (**d**) is SPR propagation length.

A surface plasmon (SP) consists of an evanescent wave field, whose resonance component is absorbed by free electrons contained in the thin metal film, as shown in Figure 1. Figure 1a illustrates the electromagnetic field configuration excited by a plane wave of incident amplitude impinging on the metal layer from the dielectric at an angle of incidence. We measured the SPR reflectivity curves for an anti-symmetrically structured SPR device, *i.e.*, a glass-dielectric-metal-dielectric (test fluid medium) interface. The SP modes of these anti-symmetrically structured SPR devices were excited by irradiating both sides of the Au film, which changed the incidence angle (*θ*_2_ < *θ*_1_) and the momentum shift (*k_x_*_2_ < *k_x_*_1_) at the Au/ZnO interfaces. Therefore, the SPR devices will be changed less than the FWHM of the SPR reflectivity curve leading to a longer propagation length at the Au/ZnO interface. In general, the metal films in SPR devices are made of Au because of its excellent chemical resistance and high extinction coefficient (*k*). As shown in Figure 1b, Cr is highly reflective and has a high extinction coefficient (*k*) [28,29]. Similar to the intermediary layers for long-range surface plasmons (LRSPs) [30–32], our design of anti-symmetrically structure of low-loss surface plasmon resonance (LLSPR) exhibits symmetric electric field (Ez) on both sides of the Au layer and thus leads to the reduced damping loss. In our previous studies, we have used these details for obtaining the dielectric structure results [16]. LLSPR and LRSPs technologies have the same features, such as longer surface propagation lengths, higher electric field strengths, and sharper angular resonance curves than conventional surface plasmons. Similar conclusions have been proposed by Warket *et al.* [33] and Patskovskyet *et al.* [34]. In addition, we explained from the basic surface plasmon resonance characteristics. We then naturally obtain a complex parallel wavenumber ${\text{k}}_{\text{SP}}={\text{k}}_{\text{SP}}^{\prime }+{\text{ik}}_{\text{SP}}^{\prime \prime }$. The real part ${\text{k}}_{\text{SP}}^{\prime }$ determines the SPP wavelength, while the imaginary part ${\text{k}}_{\text{SP}}^{\prime \prime }$ accounts for the damping of the SPP as it propagates along the interface, from the SP dispersion Equations (1) and (2) [1,35]:

(1) $${\text{k}}_{\text{SP}}=\text{k}\sqrt{\frac{{ɛ}_{\text{d}}\left({ɛ}_{\text{m}}^{\prime }+{\text{i}ɛ}_{\text{m}}^{\prime \prime }\right)}{{ɛ}_{\text{d}}+\left({ɛ}_{\text{m}}^{\prime }+{\text{i}ɛ}_{\text{m}}^{\prime \prime }\right)}}={\text{k}}_{\text{SP}}^{\prime }+{\text{ik}}_{\text{SP}}^{\prime \prime }$$

(2) $${\text{k}}_{\text{SP}}^{\prime }=\text{k}{\left(\frac{{ɛ}_{\text{d}}{ɛ}_{\text{m}}^{\prime }}{{ɛ}_{\text{d}}+{ɛ}_{\text{m}}^{\prime }}\right)}^{\overset{1}{\;}/\underset{2}{\;}},{\text{k}}_{\text{SP}}^{\prime \prime }=\text{k}{\left(\frac{{ɛ}_{\text{d}}{ɛ}_{\text{m}}^{\prime }}{{ɛ}_{\text{d}}+{ɛ}_{\text{m}}^{\prime }}\right)}^{\overset{3}{\;}/\underset{2}{\;}}\cdot \frac{{ɛ}_{\text{m}}^{\prime \prime }}{2{\left({ɛ}_{\text{m}}^{\prime }\right)}^{2}}$$

(3) $$\delta_{\text{SP}}=\frac{1}{2{\text{k}}_{\text{SP}}^{\prime \prime }}=\frac{\text{c}}{\omega }{\left(\frac{{ɛ}_{\text{d}}+{ɛ}_{\text{m}}^{\prime }}{{ɛ}_{\text{d}}{ɛ}_{\text{m}}^{\prime }}\right)}^{\frac{3}{2}}\cdot \frac{{\left({ɛ}_{\text{m}}^{\prime }\right)}^{2}}{{ɛ}_{\text{m}}^{"}}$$

Here δ_sp_ is propagation length, which can be identified by the imaginary part, k″_SP_, of the complex surface plasmon wavevector. SPR resonance width and propagation length were influenced by the imaginary part (k″_SP_). Figures 1c,d show the imaginary part in surface electric field resonance width with propagation length relation. The propagation length of the SPP along the interface is determined by k″_SP_, which is responsible for an exponential damping of the electric field intensity. The exponential decay length of the electric field is 1/(2k″_SP_) for the intensity. The relationship between the electric field intensity and propagation length can be expressed as ${\left|\text{E}\right|}^{2}\propto {\text{e}}^{-2{\text{k}}_{\text{SP}}^{\prime \prime }\text{x}}$. This illustrates their sensitivity to surface properties.

### 2.2. Materials

We determined the optimal thickness for a ZnO thin film at which its refractive index and the FWHM of the SPR reflectivity curve decreased. As compared to conventional SPR devices, these anti-symmetrically structured SPR devices showed a considerably narrower SPR reflectivity curve measured by irradiating a 830 nm laser light source through an SF10 prism substrate (refractive index *n* = 1.72, 3 × 3 cm^2^, 60° angle, Edmund Optics, Inc. Barrington, NJ, USA) with an index-matching oil (*n* = 1.72 ± 0.005, R.P. Cargille Laboratories, Inc. Cedar Grove, NJ, USA) at a wavelength of 630 nm and a temperature of 25 °C. All deposited materials (ZnO, Cr, Au) used had purity >99.99%. The ethanol solutions (≥99.5%, CH_3_CH_2_OH, Sigma-Aldrich, Louis, MO, USA) used were prepared by a series dilution of ethanol in deionized water (ddH_2_O), with weight percentages (wt) of 0%, and 1.25, 2.5, 5, 10, 20, 30, 40, 50, 60, 80, and 95% ethanol solutions. The refractive indices (n) were measured using KEM RA-130 (Kyoto Electronics, Kyoto, Japan), a refraction meter. The obtained n values at room temperature while the concentration of contacting ethanol increased from 0 to 95 wt%, which corresponded to an increase from 1.33128 to 1.44. The results demonstrated that the bilayered (ZnO/Au) metal films produce a sharper SPR dip profile than pure Au films and retain the high chemical stability of Au films. However, the higher chemical stability of gold has resulted in a wider preference for this noble metal for biosensing applications.

### 2.3. Fabrication of the Intermediary Layer

In order to find the optimum conditions under which an SPR device shows a narrow SPR reflectivity curve, we fabricated eight different thin-film devices: six ZnO/Au devices, a Cr/Au device, and an ITO/Au device. The fabrication parameters of the eight devices are listed in Table 1.

To analyze the growth orientation of the ZnO (002) crystals, we fabricated four different thin films for (ZnO/Au)-1, (ZnO/Au)-2, (ZnO/Au)-3, and (ZnO/Au)-4 devices, at different substrate temperatures (°C) using radio-frequency (RF) power (watt, W). The (ZnO/Au)-4 is more about different gold thickness rather than ZnO growth condition, because it has the same condition as the (ZnO/Au)-1. Then to optimize the ZnO film thickness, we fabricated three ZnO films with different thicknesses at 200 °C and RF power of 200 W for (ZnO/Au)-1, (ZnO/Au)-5, and (ZnO/Au)-6 devices. We used high temperature and high RF power to significantly improve the adsorbability of the ZnO within the Au film. The ZnO films were grown on SF10 prism substrates using a 13.56-MHz RF sputtering system. A metallic Zn (99.99%) target was used for ZnO deposition. A working pressure of 3 mTorr was used during the deposition, and the working gas was a mixture of Ar and O_2_ at a 4:3 ratio. For comparison, we fabricated conventional SPR devices with (Cr/Au)-7 and (ITO/Au)-8 thin films. The (Cr/Au)-7 device consisted of a 2-nm Cr (99.9%) layer deposited using an electron beam evaporator. The (ITO/Au)-8 devices consisted of a 0.7 mm glass substrate and a 200 nm ITO thin film with a sheet resistance of 46.6 Ω/sq (Merck Display Tech. Ltd., Taiwan). Next, 50 nm Au (99.99%) films were deposited using an electron beam evaporator in a vacuum of approximately 3 × 10^−6^ Torr at an evaporation rate of approximately 0.2 Å/s.

## 3. Results and Discussion

### 3.1. Comparisons of ZnO Thin Films for Optimizing the Design of Anti-Symmetrically Structured SPR Devices

**Figure 2.**
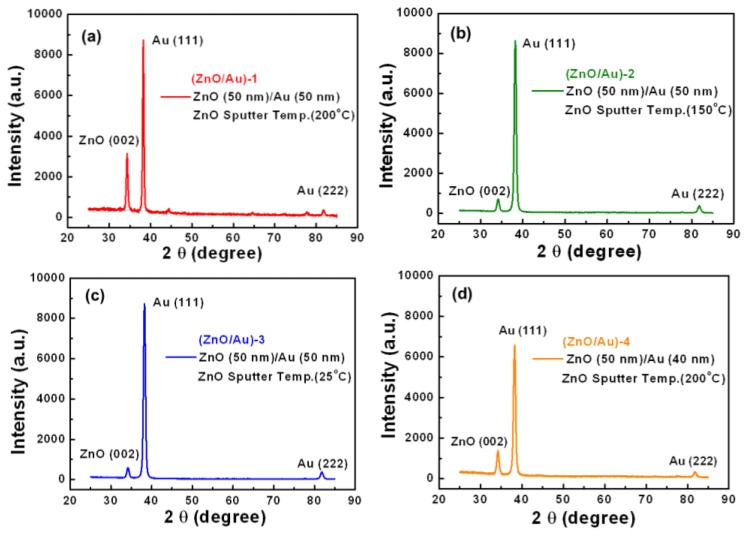

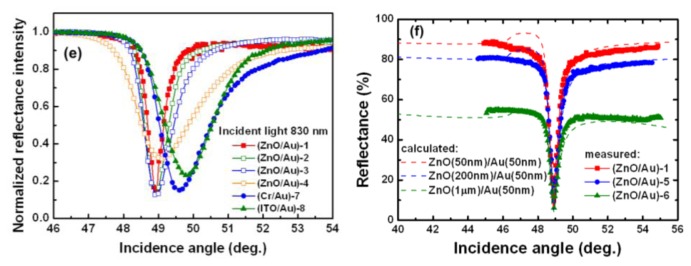
XRD patterns of ZnO thin films deposited on SF10 substrates under different conditions. Diffraction angle (2θ), FWHM, and XRD peak intensity for (**a**) (ZnO/Au)-1, (**b**) (ZnO/Au)-2, (**c**) (ZnO/Au)-3, and (**d**) (ZnO/Au)-4; (**e**) SPR reflectivity curves of (ZnO/Au)-1, (ZnO/Au)-2, (ZnO/Au)-3, (ZnO/Au)-4, (Cr/Au)-7 and (ITO/Au)-8, (**f**) Theoretically calculated (line) and measured (symbols) reflectance SPR spectra of (ZnO/Au)-1, (ZnO/Au)-5 and (ZnO/Au)-6 devices in water for a light source with a wavelength of 830 nm.

We compared the properties of the ZnO (002) thin film with *c*-axis oriented crystals in the (ZnO/Au)-1, (ZnO/Au)-2, (ZnO/Au)-3, and (ZnO/Au)-4 devices to determine the optimal design. Figures 2a–d show X-ray diffraction (XRD) patterns (Nonius, Kappa CCD Single-crystal XRD) for ZnO/Au thin films deposited on SF10 prism substrates. We analyzed the anti-symmetric structures of the (ZnO/Au) devices and evaluated their performances. The XRD patterns show three peaks corresponding to the (002) plane of ZnO and (111) and (222) planes of the Au films. The ratio between the maximum intensities of the (002) diffraction peak was (ZnO/Au)-1:(ZnO/Au)-2:(ZnO/Au;-3 = 5.4:1.2:1.

For refractive index measurement, the EP^3^ imaging ellipsometer (Nanofilm Technologie GmbH, Göttingen, Germany) was used to observe the circularly polarized light reflected from a sample. The wavelengths of the incident laser light source were 830, 643, and 532 nm. From Table 2, it can be seen that the refractive index of the thin-film layers depended on the wavelength of the incident light. The (ZnO/Au)-1 device showed high refractive indices of 1.97, 1.97, and 1.98 at wavelengths of 830, 643, and 532 nm, respectively. The refractive indices of (ZnO/Au)-1, (ZnO/Au)-2, and (ZnO/Au)-3 devices at a wavelength of 830 nm are 1.97, 1.95, and 1.94, respectively. These results show a positive relationship between an increase in RF power and substrate temperature, which can dramatically affect the optical refractive index of ZnO thin films. The variation in the refractive index can be attributed to changes in the growth density of the ZnO thin film [36–38].

Figure 2f shows the reflectance spectra of the (ZnO/Au)-1, (ZnO/Au)-5 and (ZnO/Au)-6 experimentally measured and theoretically calculated on the sensitivity curve for the thick ZnO layer using Fresnel's law, at an incident wavelength of 830 nm. Our experimental results showed that the large thicknesses of the ZnO film, could affect the FWHM and effective coupling of SPR curve.

We measured and compared the (002) diffraction peak intensities and the FWHM of the SPR reflectivity curves for four different ZnO/Au devices, a Cr/Au device, and an ITO/Au device. Figure 2e shows that the SPR reflectivity curve of (ZnO/Au)-1 is narrower than those of (ZnO/Au)-2, (ZnO/Au)-3, and (ZnO/Au)-4. The 40-nm-thick Au film in the (ZnO/Au)-4 device has a slightly larger FWHM of 2° compared to that of the other samples. The FWHM of the SPR reflectivity curve of the (ZnO/Au)-1, (ZnO/Au)-2, (ZnO/Au)-3, (Cr/Au)-7 and (ITO/Au)-8 devices were 0.551°, 0.779°, 0.898°, 1.309°, and 1.191°, respectively. The dependence of the dielectric properties of the ZnO thin film on temperature was studied in the range from 25 to 200 °C; the corresponding decrease in the FWHM values of the SPR reflectivity curves at these temperatures was from 0.898° to 0.551°. Our results showed that ZnO thin films fabricated at high RF power and high temperature had a good single-crystal, hexagonal structure. High quality ZnO films can be grown at substrate temperatures above 200 °C. The results show that the SPR reflectivity curve of the (ZnO/Au)-1 device exhibited a small FWHM value. However, the quality of the ZnO (002) crystal was found to be the most important parameter that relates the thin-film density to the FWHM of the SPR reflectivity curve.

### 3.2. Theoretical Analysis and Verification

For theoretical analysis and verification of our results, the fabrication parameters of our anti-symmetrically structured SPR devices were calculated using Fresnel's equations of multilayer theory [39]. The SPR reflectance was measured at incident angles of 46–56° using Fresnel's equation for a four-layer (three-interface) system. The four layers (0, 1, 2, and 3) correspond to the SF10 substrate, intermediary layer, Au film, and test fluid medium, respectively. We used the refractive index (*n*) and extinction coefficient (*k*) values of the Au, ZnO, Cr, and ITO films, measured at a wavelength of 830 nm, to obtain ε_Au_ = −29.33 + 2.052*i*, ε_ZnO_ = 3.84 + 0*i*, ε_Cr_ = 0.87 + 37.84*i*, and ε_ITO_ = 2.34 + 0*i*, respectively.

**Figure 3.**
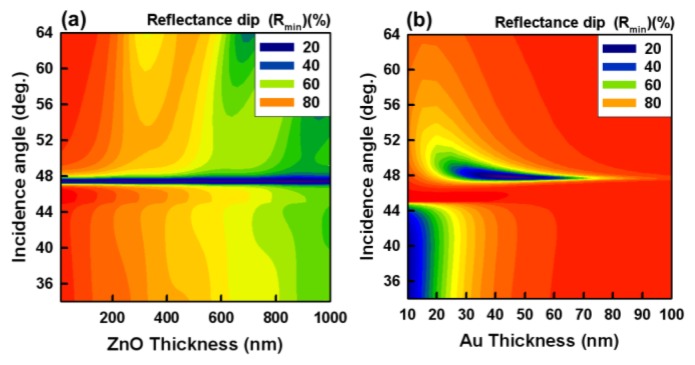
(**a**) Theoretically calculated reflectance SPR spectra of devices in water for a light source with a wavelength of 830 nm. (**b**) Theoretically calculated values at an excitation wavelength of 830 nm showing the dependence of the SPR reflectivity curve of the *in situ* (ZnO/Au) devices as a function of Au thickness at a fixed ZnO film thickness of 50 nm.

We analyzed the relationship between the FWHM of the SPR reflectivity curve and the ZnO thin film thickness. To evaluate the sensitivity of SPR sensors, the most important parameters are FWHM, minimum reflectance (*R_min_*), resolution and resonant angle shift. Narrow and sharp SPR reflectivity curves can be produced by making a smooth surface of the Au film, which can minimize the error in determining the SPR resonant angle. Figure 3a shows the reflectance spectra of the intermediary ZnO layers calculated using Fresnel's law, at an incident wavelength of 830 nm and a fixed Au layer thickness of 50 nm. The *R_min_* values of the calculated ones are approximately 48.4° with the reflectance of 5%. Results showed that *R_min_* can be very close to zero when metal layer with appropriate thickness is deposited. From the results, a ZnO film with thickness of 50 nm performed the best sensitivity, and a ZnO thin film thickness of less than 30 nm resulted in incomplete coverage of the relatively rough substrate surfaces. The roughness effect of the ZnO film on the sensitivity of SPR sensor chips is analyzed theoretically and experimentally. It can be concluded that the sensitivity performance of SPR sensor chip can be improved by the surface roughness conditions of the ZnO films. Our results showed that the large thicknesses of the ZnO film could affect the FWHM and effective coupling of the SPR reflectivity curve. The optimal thickness of ZnO film ranges from 50 to 100 nm.

To determine the optimal Au thickness, we calculated the SPR intensity and FWHM as functions of Au thickness. We set two control conditions: a fixed ZnO layer thickness of 50 nm, and an incident wavelength of 830 nm. The calculations showed that the reflectance dip and FWHM were the lowest at a Au thickness of 45 nm, as shown in Figure 3b. The FWHM of the SPR reflectivity curve has an error margin of ±0.2°. From these results, we infer that the optimal thickness of the Au film in the (ZnO/Au) devices was approximately 50 nm. We found that to observe an extremely narrow dip in the SPR reflectivity curve, which is associated with the excitation of the low-loss-plasmon in an anti-symmetric structure, scanning across the angles of incidence of the input light had to be carried out with a very high sensitivity.

**Figure 4.**
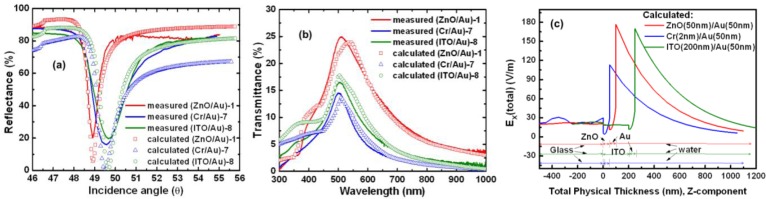
(**a**) Theoretically calculated (symbols) and measured (solid lines) reflectance SPR spectra of devices at a wavelength of 830 nm in below water. (**b**) Measured (solid lines) *vs.* theoretically calculated (symbols) transmittance spectra of fabricated devices, in water. (**c**) Intensity of the electric field (V/m) amplitude through the dielectric structure when the incident angle is the SPR angle. The plasmon electric field intensity is the highest with maximum coupling of the incident excitation light at a wavelength of 830 nm. The high electric field enhancement on the metal surface due to SPPs has been exploited in the sensing of biochemical molecules.

Figure 4a shows the measured and calculated SPR angles obtained with their FWHM values. The calculated SPR angles for the (ZnO/Au)-1, (Cr/Au)-7, and (ITO/Au)-8 devices were 48.9°, 49.5°, and 49.6°, respectively (the measured SPR angles under water were 48.914°, 49.49°, and 49.655°, respectively). The calculation results showed that the SPR reflectivity curve of the (ZnO/Au)-1 device exhibited an FWHM value of 0.56°. This FWHM value is less than those of the (Cr/Au)-7 and (ITO/Au)-8 devices, which were 1.2° and 1.05°, respectively. These SPR results are compared in Table 3.

However, we found discrepancies between the measured and calculated values of the SPR dip, probably because the grain size effect on the surface, rough boundaries, and other parameters of the metal layers being ignored in the theoretical calculation [40]. Optical transmittance spectra were recorded at room temperature using a U-2900 ultraviolet-visible-near infrared (UV-vis-NIR) double-beam spectrophotometer (Hitachi High-Technologies Corporation, Tokyo, Japan) over a wavelength range of 300–800 nm and at an incident angle of 0° (normal angle). Figure 4b shows the measured and calculated transmittance spectra of the (ZnO/Au)-1, (Cr/Au)-7, and (ITO/Au)-8 devices. The transmittance spectra of the (ZnO/Au)-1, (Cr/Au)-7, and (ITO/Au)-8 devices show peaks at 513, 501, and 506 nm, respectively; all these peaks fall within the wavelength range of green light. The (ZnO/Au)-1 device showed higher transparency compared to the (Cr/Au)-7 and (ITO/Au)-8 devices. The (Cr/Au)-7 and (ITO/Au)-8 devices exhibited lower transparency owing to high reflection and absorption by the intermediary Cr [29] and ITO [41–43] layers. In the visible light region, the average transmittance by the ITO and ZnO thin films was approximately 80% and 90%, respectively. In each case, the transmission coefficient in Fresnel's equations and Snell's Law is given by *T* = 1 – *R* – *A*, where *T* represents transmission, *A* is absorbance, and *R* is reflection [16,39]. When the transmission through the ZnO/Au interfaces reaches its highest value, the intensity of the electromagnetic field reaches its maximum on the surface [44]. For the measurements and calculations, we assumed that the incident light is normal to the interface (*θ_i_* = *θ_t_* = 0). The results showed that the calculated values (symbols) were close to the values (solid line) measured from the transmittance spectra.

Theoretical calculations of the effects of the plasmon resonance angle on the interface electric field of the (ZnO/Au)-1, (Cr/Au)-7, and (ITO/Au)-8 devices are shown in Figure 4c. The longitudinal electric field in a thin metal film with an anti-symmetric SP mode and the resulting low-loss energy propagation depend on the dielectric load of the ZnO layer. The objective of this effort is to determine, by calculating the planar electric field potential and the attractive potential, the total field energy propagation (*ΔE*) which can be obtained approximately as follows Equation (4) [45–47]:

(4) $$\Delta E=2\text{v}\int_{Z_{tp}}^{\infty }\frac{dE}{dx}\left(z\right)\frac{1}{\left[\nu_{z}(z)\right]}dz$$

where *Z_tp_* and *v_z_(z)* denote the turning point and the value of the component of the velocity normal to the surface, respectively, which both depend on the SPR angle. The calculation results show that the (ZnO/Au)-1 device had a maximum electric field and a large propagation length at the ZnO/Au and Au/water interfaces. An enhanced electric field intensity is generated around the ZnO layers due to the SPR effect of the metal structure, and the maximum electric field intensity is increased from 107 to 180 V/m due to the anti-symmetrically structured effect of the SPR devices. We observed that the ratio of the total field energy propagation intensity (*ΔE_x_*) of the devices was (ZnO/Au):(ITO/Au):(Cr/Au) = 1.7:1.5:1. Because of its high refractive index, an anti-symmetric structure results in a greater sensitivity of the electric field in the interface region. It should be noted that the longitudinal electric field distribution of the anti-symmetric electric field mode, such as that in LRSPs [30–32], on both sides of the Au layer is inversely proportional to the reduced damping loss. This electric field propagation is usually interpreted as a consequence of the spatial compression at the Au film decays exponentially and limits the sensing depth to approximately 1 μm, as shown in Figure 4c. However, we see a clean example of the anti-symmetric structure in the Kretschmann geometry (Figure 1a) in the case of ideal plane interfaces, *i.e.*, it causes a narrower SPR resonance peak. In addition, the illumination field plays an important role for optimized transmission through an intermediary ZnO layer.

### 3.3. Evaluation of the Detection Sensitivity for SPR Devices

**Figure 5.**
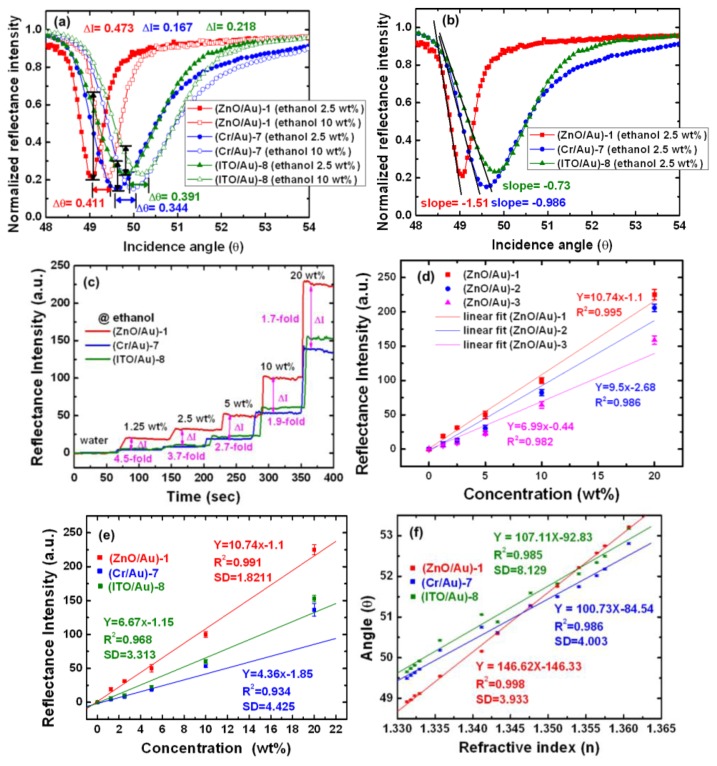
Comparison of SPR reflectivity curves obtained at different ethanol concentrations. (**a**) Reflective intensity and SPR angle shift. (**b**) Analysis of the change in slope of the SPR reflectivity curves. (**c**) Real-time monitoring of the response of (ZnO/Au)-1, (Cr/Au)-7, and (ITO/Au)-8 devices in water (0 wt%) and in 1.25, 2.5, 5, 10, and 20 wt% ethanol solutions. (**d**) Intensity interrogation mode measurement results obtained for the (ZnO/Au)-1, (ZnO/Au)-2 and (ZnO/Au)-3 devices in 0–20 wt% ethanol solutions. (**e**) Intensity interrogation mode measurement results obtained for linear regression of the (ZnO/Au)-1, (Cr/Au)-7 and (ITO/Au)-8 devices over dynamic sensing range of 0–20 wt%. (**f**) Angular interrogation mode measurement results obtained for the (ZnO/Au)-1 device in water and ethanol solutions; the angle shifted from 48.914° in water to 53.217° in 95 wt% ethanol. Calibration curves for (ZnO/Au)-1, (Cr/Au)-7, and (ITO/Au)-8 devices in water and ethanol (1.25, 2.5, 5, 10, 20, 30, 40, 50, 60, 70, 80, and 95 wt%).

SPR reflectivity curves were measured in the angular interrogation mode using an EP^3^ imaging system (Nanofilm Technologie GmbH). A high flow rate (40 μL/min) was used to minimize mass transport effect. The SPs were excited in the Kretschmann prism-coupling configuration using 830 nm light, and the incident angle was varied from 45°–65°, depending on the type of metal and the composition of the test fluid medium. When the ethanol concentration was increased from 2.5 to 10 wt%, the SPR angle shifted from 49.063° to 49.474°, from 49.63° to 49.974°, and from 49.815° to 50.206° for (ZnO/Au)-1, (Cr/Au)-7, and (ITO/Au)-8, respectively. The corresponding reflectance intensity changes (Δ*I*) were 0.473, 0.167, and 0.218 (a.u.) for (ZnO/Au)-1, (Cr/Au)-7, and (ITO/Au)-8, respectively (Figure 5a). Another important difference among these three devices was the slope (*dI*/*dθ*) of the SPR reflectivity curve. A steep slope represents high sensitivity of the SPR sensor. The calculated slopes, shown in Figure 5b, were −1.51, −0.986, and −0.73 for (ZnO/Au)-1, (Cr/Au)-7, and (ITO/Au)-8, respectively. These SPR results are compared in Table 3.

The real-time reflectance intensity was measured in the intensity interrogation mode of the SPR. We used an imaging system (GWC Technologies Inc., Madison, WI, USA) with a Kretschmann prism-coupling configuration. The intensities of 790-nm light reflected at a fixed angle of 49° were measured. This method enabled real-time detection of ethanol. The change in reflection intensity was recorded as the difference in the lowest points on the SPR reflectivity curve. Ethanol was diluted with deionized water to obtain concentrations ranging from 0 to 20 wt%. A high flow rate (40 μL/min) was used to minimize mass transport effects.

Figure 5c shows the performance of SPR devices in ethanol solutions at a concentration of 0 (deionized water), 1.25, 2.5, 5, 10, and 20 wt%. A comparison of the real-time ethanol signals showed that the (ZnO/Au)-1 device (red line) had a shorter reaction time and higher steady-state response compared to the conventional (Cr/Au)-7 (green line) and (ITO/Au)-8 (blue line) devices.

A comparison between the measured reflectance intensity shifts for the (ZnO/Au)-1 and (Cr/Au)-7 devices in 1.25, 2.5, 5, 10 and 20 wt% ethanol solutions revealed relative 4.5-, 3.7-, 2.7-, 1.9-, and 1.7-fold increases in the reflectance intensity. The narrower SPR dip of the (ZnO/Au)-1 device has a higher signal-to-noise ratio (SNR) of 9:1. The (ZnO/Au)-1 device is exhibited to hold both a narrow SPR reflectivity curve and a large response to the changes in the refractive index, which improve its sensitivity in both SNR and detection limit. The resolution of the intensity interrogation mode can be calculated using the following equation [48]:

(5) $$\delta_{\text{res}}=\left(\Delta_{n}/\Delta_{I}\right)\delta_{g}$$

where *δ_res_* is the resolution in terms of RIU, Δ*_n_* and Δ*_I_* are the changes in the refractive index and the corresponding resonance intensity, respectively, and *δ_g_* is the 8 bits/pixel (= 255 grayscales) dynamic range of the CCD imaging system in the GWC-SPR instrument. The (ZnO/Au)-1 device could detect ethanol concentrations from 0 (1.33128 RIU) to 20 wt% (1.34117 RIU) [49]. When the ethanol concentration was over 20 wt%, the intensity response reached saturation, and the reflectance intensity changed from 0.21 to 0.946 (a.u.). On the basis of the Δ*_n_* and Δ*_I_* values obtained, resolutions in our measurement system were found to be 5.25, 10.11, and 9.88 × 10^−5^ RIU for the (ZnO/Au)-1, (Cr/Au)-7, and (ITO/Au)-8 devices, respectively.

Figure 5d shows that the experimentally determined anti-symmetric structure (ZnO/Au) device characteristics in intensity interrogation mode exhibited a good agreement with the calibration curve. The average signal reflectance intensity of (ZnO/Au)-1, (ZnO/Au)-2, and (ZnO/Au)-3 devices at each step was plotted as a function of the concentration. The anti-symmetric structure devices gave a linear plot for the range of 0 to 20 wt%, and the regression equations of the slope of each fitting curve were the following: 10.74 for the (ZnO/Au)-1 device, 9.5 for the (ZnO/Au)-2 device, and 6.99 for the (ZnO/Au)-3 device. The resolutions in our measurement system were 5.25, 6.4, and 7.95 × 10^−5^ RIU for the (ZnO/Au)-1, (ZnO/Au)-2, and (ZnO/Au)-3 devices, respectively. The results obtained show that the optimal device of (ZnO/Au)-1 have the high resolution and slope of intensity mode.

Figure 5e shows that the measured SPR characteristics in the intensity interrogation mode are in good agreement with the calibration curve. The (ZnO/Au)-1 device showed a linear plot for ethanol concentrations ranging from 0 to 20 wt%, according to a linear regression equation *y* = 10.74*x* – 1.1 and a correlation coefficient (*R*^2^) of 0.991, where *y* represents the reflectance intensity (a.u.), and *x* is the ethanol concentration. In addition, as seen in Table 3, the linear regression equations of the calibration curves for the (Cr/Au)-7 and the (ITO/Au)-8 device were *y* = 4.36*x* − 1.85 with a correlation coefficient *R*^2^ = 0.934 and *y* = 6.67*x* − 1.15 with a correlation coefficient *R*^2^ = 0.968, respectively.

The SPR response characteristics in the angular interrogation mode of the (ZnO/Au)-1 device for deionized water (0 wt%), and for 1.25, 2.5, 5, 10, 20, 30, 40, 50, 60, 80, and 95 wt% ethanol solutions; the refractive indices were obtained from the literature [49]. The resolution in the angular interrogation mode can be calculated as:

(6) $$\delta_{\text{res}}=\left(\Delta_{n}/\Delta_{\theta }\right)\delta_{A}$$

where Δ*_n_* and Δ*_θ_* are the changes in the refractive index and the corresponding resonance angle, respectively, and *δ_A_* is the angular resolution of 10^−3^, of the EP^3^ imaging instrument. From the measured values of Δ*_n_* and Δ*_θ_*, the resolution of our system was calculated as 6.76, 9.78, and 9.37 × 10^−6^ RIU for the (ZnO/Au)-1, (Cr/Au)-7, and (ITO/Au)-8 devices, respectively (Table 3).

Figure 5f shows that the SPR responses to the corresponding average inaccuracy of SPR angles were ±0.018°, ±0.021°, and ±0.021° (*n* = 3) for the (ZnO/Au)-1, (Cr/Au)-7, and (ITO/Au)-8 devices, respectively. These results were in good agreement with the calibration curves. The linear regressions of the calibration curves were *y* = 146.62*x* − 146.33 (correlation coefficient, *R*^2^ = 0.998) for (ZnO/Au)-1, *y* = 100.73*x* − 84.54 (correlation coefficient, *R*^2^ = 0.986) for (Cr/Au)-7, and *y* = 107.11*x* − 92.83 (correlation coefficient, *R*^2^ = 0.985) for (ITO/Au)-8, where *x* is the RIU and *y* is the SPR angle (*θ*). For the (ZnO/Au)-1 device, the linear range of detection for the ethanol solutions was 0–95 wt%; the data show that this device exhibited a little angle shift, and higher sensitivity compared to the other devices. The error bar indicates the standard deviation (SD) of 10 measurements, which were taken for 1 min; the SD value were 3.933, 4.003, and 8.129 for (ZnO/Au)-1, (Cr/Au)-7, and (ITO/Au)-8 devices, respectively. The fitted curve of RIU sensitivity signal is seen to be almost proportional to the refractive index, and the gradient of the line of the (ZnO/Au)-1, (Cr/Au)-7, and (ITO/Au)-8 devices were 147.958, 102.227, and 106.77 degree per RIU, respectively.

**Figure 6.**
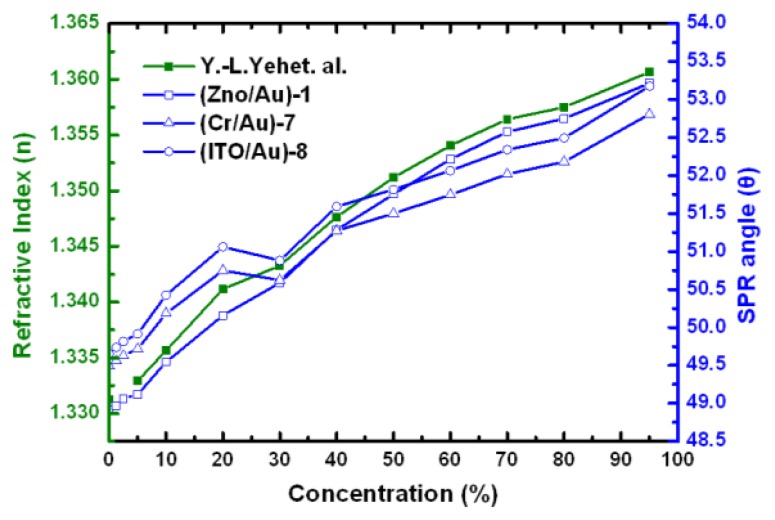
Comparison of the linear relationships between concentrations and refractive index and between concentrations and SPR angle.

Optical methods offer the potential to obtain wider dynamic range and higher sensitivity in the specified concentration range by either angle or intensity difference to the refractive index change. Figure 6 shows the SPR measurements and the calibration curve obtained for deionized water (0%), 2.5%, 10%, 20%, 40%, 60%, 80%, and 95% ethanol concentrations (as weight percentages). Putting our data together with Yeh's data [49], we can find that both methods follow the similar linear trend as shown in Figure 6. The (ZnO/Au)-1 chip provides a larger angle shift than the conventional SPR (Cr/Au)-7 and (ITO/Au)-8 chips and thus higher sensitivity.

## 4. Conclusions

The use of ZnO intermediary layers in anti-symmetric structures enabled the optimization of SPR biosensor design and efficiency. It was found that the high refractive index and high transparency of ZnO lead to a large propagation length at the Au/ZnO interface. The narrowing of the SPR reflectivity curve is expected to provide increased sensitivity, because a narrower SPR dip can be localized with a higher precision. We varied the SPR device fabrication parameters to demonstrate that a higher-quality ZnO (002) crystal can enhance SPR sensitivity. The ratio between the maximum intensities of the (002) diffraction peak was (ZnO/Au)-1:(ZnO/Au)-2:(ZnO/Au)-3 = 5.4:1.2:1, and the FWHM values of the SPR reflectivity curves of the (ZnO/Au)-1, (ZnO/Au)-2, (ZnO/Au)-3 devices were 0.551°, 0.779°, 0.898°, respectively. The resolutions of the intensity interrogation modes were 5.25, 6.4, and 7.95 × 10^−5^ RIU for the (ZnO/Au)-1, (ZnO/Au)-2, and (ZnO/Au)-3 devices, respectively. SPR sensor chips that produce sharp SPR curves and large angle shifts represent a better sensitivity. Experimental results show that FWHM were 1.2 degree for (Cr/Au)-7 and 0.56 degree for (ZnO/Au)-1 device. The results showed that the (ZnO/Au)-1 device had the maximum electric field and a large propagation length at the ZnO/Au and Au/water interfaces. The (ZnO/Au)-1 device exhibited a wider linearity range and much higher sensitivity for the detection of ethanol at a low concentration. In our future work, we will explore the feasibility of this approach for the development of high-performance biosensors that could provide a crucial tool for detecting small molecule–target drug interactions and toxic chemical substances.

## Figures and Tables

**Table 1.** Fabrication parameters for the eight devices.

**Devices**	**Intermediary Layer**	**S.T. (°C)** ^a^	**Power (w)** ^b^	**Au film**
(ZnO/Au)-1	50 nm	200	200	50 nm
(ZnO/Au)-2	50 nm	150	150	50 nm
(ZnO/Au)-3	50 nm	25	150	50 nm
(ZnO/Au)-4	50 nm	200	200	40 nm
(ZnO/Au)-5	200 nm	200	200	50 nm
(ZnO/Au)-6	1 μm	200	200	50 nm
(Cr/Au)-7	2 nm			50 nm
(ITO/Au)-8	200 nm			50 nm

a S.T. is the substrate temperature (°C);

b The RF sputtered power supply unit is watt (W).

**Table 2.** Comparison between refractive indices of (ZnO/Au)-1, (ZnO/Au)-2, and (ZnO/Au)-3 devices measured at wavelengths of 830, 643, and 532 nm, respectively, measured at room temperature.

**λ**	**ZnO thin films (n & k)**		

	**(ZnO/Au)-1**	**(ZnO/Au)-2**	**(ZnO/Au)-3**
830 nm	1.97 & 0	1.95 & 0	1.94 & 0
643 nm	1.97 & 0	1.96 & 0	1.94 & 0
532 nm	1.98 &0	1.96 & 0	1.95 & 0

1. n & k are the refractive index and extinction coefficient, respectively; 2.The error margins in the refractive index and extinction coefficient of the thin films are ±0.02.

**Table 3.** Performance comparisons of (ZnO/Au)-1, (Cr/Au)-7, and (ITO/Au)-8 devices.

	**(ZnO/Au)-1**	**(Cr/Au)-7**	**(ITO/Au)-8**
Measured SPR (FWHM) (°)	0.551	1.309	1.191
Calculated SPR (FWHM) (°)	0.56	1.2	1.05
Δ (I) (a.u.), ethanol (2.5%–10%) (normalized)	0.473	0.167	0.218
Δ (θ) (deg.), ethanol (2.5%–10%)	0.411	0.344	0.391
Intensity slope	−1.51	−0.986	−0.73
Dynamic range (Δn, RIU) of intensity mode	1.33128–1.34117	1.33128–1.3512	1.33128–1.35
ΔI of intensity shift (a.u.)	0.21–0.946	0.18–0.95	0.21–0.95
Resolution of intensity mode (×10^−5^ RIU) ^a^	5.25	10.11	9.88
Dynamic range (Δn, RIU) of angle mode	1.33128–1.44	1.33128–1.483	1.33128–1.475
Δθ of angle shift (degree)	48.914–65	49.49–65	49.655–65
Resolution of angle mode (×10^−6^ RIU) ^b^	6.76	9.78	9.37

SPR (FWHM) is the FWHM value of the SPR absorbance curve; Δ (I) (a.u) is the SPR reflectance intensity shift; Δ (θ) (deg.) is the SPR reflectance angle shift; Intensity slope is the change in Intensity/angle (0.2°) shift; Dynamic range of angle ranges from 45° to 65°;

a Dynamic range of intensity from 0 to 255 pixels for the 8-bit CCD;

b Angular measurement with a resolution of 0.001°.
